# The Mouse Limb Anatomy Atlas: An interactive 3D tool for studying embryonic limb patterning

**DOI:** 10.1186/1471-213X-8-83

**Published:** 2008-09-15

**Authors:** April DeLaurier, Nicholas Burton, Michael Bennett, Richard Baldock, Duncan Davidson, Timothy J Mohun, Malcolm PO Logan

**Affiliations:** 1 Division of Developmental Biology, National Institute for Medical Research, The Ridgeway, Mill Hill, London, NW7 1AA, UK; 2 Institute of Neuroscience, 1254 University of Oregon, Eugene, OR 97403, USA; 3 MRC Human Genetics Unit, Western General Hospital, Crewe Road, Edinburgh, EH4 2XU, Scotland, UK

## Abstract

**Background:**

The developing mouse limb is widely used as a model system for studying tissue patterning. Despite this, few references are available that can be used for the correct identification of developing limb structures, such as muscles and tendons. Existing textual references consist of two-dimensional (2D) illustrations of the adult rat or mouse limb that can be difficult to apply when attempting to describe the complex three-dimensional (3D) relationship between tissues.

**Results:**

To improve the resources available in the mouse model, we have generated a free, web-based, interactive reference of limb muscle, tendon, and skeletal structures at embryonic day (E) 14.5 . The Atlas was generated using mouse forelimb and hindlimb specimens stained using immunohistochemistry to detect muscle and tendon. Limbs were scanned using Optical Projection Tomography (OPT), reconstructed to make 3D models and annotated using computer-assisted segmentation tools in Amira 3D Visualisation software. The annotated dataset is visualised using Java, JAtlasView software. Users click on the names of structures and view their shape, position and relationship with other structures within the 3D model and also in 2D virtual sections.

**Conclusion:**

The Mouse Limb Anatomy Atlas provides a novel and valuable tool for researchers studying limb development and can be applied to a range of research areas, including the identification of abnormal limb patterning in transgenic lines and studies of models of congenital limb abnormalities. By using the Atlas for "virtual" dissection, this resource offers an alternative to animal dissection. The techniques we have developed and employed are also applicable to many other model systems and anatomical structures.

## Background

The mouse is a frequently used model for studying limb development and the anatomical similarities between mouse and human limbs make it an excellent system for understanding human limb defects. An advantage of the mouse model is that many molecular techniques have been established for studying the genetic pathways regulating limb development, so it is possible to link genetic mechanisms with patterning events. Typically, limb patterning is studied using two-dimensional (2D) microscopy of whole mount or histological sections of tissues or embryos. A significant limitation of these approaches is that they do not allow the interpretation of the complex physical relationship between individual limb elements in three-dimensional (3D) space as development proceeds. Increasingly, 3D imaging techniques, including Optical Projection Tomography (OPT), High-Resolution Episcopic Microscopy (HREM), ultrasound and ultramicroscopy are being used to study mouse and human embryonic development and anatomy [1-6]. Some of these techniques have been used to generate interactive atlases of developing organs and 3D gene expression databases (ie. EADHB [7], EMAP [8], EMAGE [9], DGEMap [10], MRIMA [11], and FishNet [12]).

Recently, we have demonstrated that 3D imaging can be used to study the embryonic mouse limb, providing an unprecedented amount of detailed information about the patterning and growth of this structure [13]. A difficulty we experienced in analysing the 3D organisation of limb tissues was the lack of accessible, intuitive resources for studying the relationship between structures. Identification of embryonic mouse limb patterning is typically based on reference books of adult mouse or rat limb anatomy [14,15]. These 2D references are challenging to apply to 3D data and are often not appropriate for interpreting embryonic structures that are not fully developed or organised in their final adult pattern.

To address this lack of resources, we have generated interactive 3D models of normal mouse embryonic limb anatomy as a reference tool for researchers studying limb development in transgenic and disease models. Our Atlas provides models of the forelimb and hindlimb of mice at E14.5 days gestation generated using OPT. Using platform-independent, Java-based, JAtlasView software [16], users navigate 3D models of limbs where individual structures are colour-coded and linked to an anatomy key. The 3D view is cross-linked to a 2D dataset, where colour-coded structures are viewed in orthogonal or user-defined sections. Unlike textual atlases, our digital atlas allows users to navigate from the ontology to the spatial representation of the embryo in 3D and 2D, and vice versa. 2D and 3D models feature over 60 individual muscles, tendons and bones linked to a colour-coded key. Stage E14.5 was chosen as this is the earliest time-point at which the individual limb elements that comprise the adult limb are easily, morphologically distinguishable.

All data and software is free for users to download. This database provides a standardisation of the names of limb structures used by biologists [17]. Feedback on the assignment of annotated structures, ease of use and robustness of software and suggestions on how this resource could be improved are welcomed. The techniques we have employed to generate the database are applicable to other model systems and anatomical structures.

## Construction and content

### Transgenic mice and immunohistochemistry

Tendons were visualised with the *Scleraxis(Scx)-GFP *reporter line on a C57Bl6 background [13,18]. Mouse embryos were staged according to Kaufman (1992) [19]. Noon on the day a vaginal plug was observed was taken as E0.5 day gestation. Embryos were harvested at E14.5, immediately exsanguinated and fixed as previously described [13]. Embryonic forelimbs and hindlimbs were skinned and stained using antibodies against *GFP *and muscle myosin as previously described [13].

### Optical Projection Tomography and segmentation

OPT scanning and 3D reconstructions of data were performed as described previously [13]. Digital scans of the specimen were used to reconstruct "virtual" transverse sections and render 3D images depicting tissues. With biological material some variation in samples is inevitable. To identify a 'standardised' E14.5 forelimb and hindlimb, a total of over 75 E14.5 limbs were analysed using various histological methods to analyse the morphology of the skeleton, muscles and tendons. Up to 15 different E14.5 forelimbs and hindlimbs were analysed by OPT scanning. Representative examples of the forelimb and hindlimb were chosen as standardised samples for the Anatomy Atlas.

Reconstructed datasets containing *GFP *(Tendon) and *Texas-Red *(Muscle) scans of *Scx-GFP *reporter forelimbs and hindlimbs were merged and converted to 8-bit greyscale to create a single dataset showing both tendon and muscle. The image stack was loaded in Amira 3D Visualisation software (version 3.1, Mercury Computer Systems, Germany) and structures of the limb were segmented and annotated using computer-assisted segmentation. Structures were assigned using semi-automated segmentation. Structures were manually outlined in every 10^th ^section in the z-axis and the automatic propagation tool in Amira was used to find the boundaries of structures between defined sections. All automatic segmentation was checked manually for accuracy and boundary adjustments were made where necessary.

Each OPT stack is 670 pixels in the z axis. The limb extends across approximately 600 pixels in each stack (equivalent to 600 of the 670 OPT sections) in the z axis. Since an E14.5 limb is approximately 8000 μm in length, we estimate optical slice thickness to be approximately 8000/600 = 13 μm. The voxel dimension is therefore estimated to be 13 × 13 × 13 μm.

### High-resolution Episcopic Microscopy (HREM)

Forelimbs and hindlimbs were dissected from exsanguinated E14.5 embryos and dehydrated overnight in 95% ethanol. Limbs were infiltrated with polymer embedding solution (JB-4 Embedding Kit, Polysciences, Warrington, PA) containing JB-4 Solution A (monomer), Benzoyl Peroxide catalyst (12.5 mg/ml) and coloured dyes Orasol black (1 mg/ml; Ciba, Macclesfield, UK), Eosin B (2.75 mg/ml; Sigma, St. Louis, MO), and Acrydine Orange (0.55 mg/ml; Sigma, St. Louis, MO) overnight at 4°C. Specimens were embedded in molds using the infiltration solution with added JB-4 Solution B (Accelerator) for polymerisation. Samples were allowed to set at room temperature overnight. Polymerised blocks were sectioned using HREM as previously described [3].

### Identification of skeletal elements, muscles, and tendons

Bones were identified from references for the adult rat [14] and adult mouse [20]. Muscles and tendons were identified using anatomical references for the adult rat [14,15,21]. Structures were positively identified on the basis of shape, position and orientation in relation to other elements. Tendons were identified as belonging to specific muscles on the basis of their myotendinous junctions. Muscles were identified on the basis of morphology, as well as associated tendon origin and insertion sites on skeletal elements.

During segmentation of the hindlimb samples, we were unable to detect a structure that could be identified as the rectus femoris muscle that is described in one reference [14] but is not illustrated in another [15]. This muscle is in a deep location so it is very unlikely to have been lost in processing. A possible reason we were unable to identify the rectus femoris muscle is that it is not a morphologically distinct muscle block in the E14.5 mouse.

### Viewing 3D models

**Figure 1:**
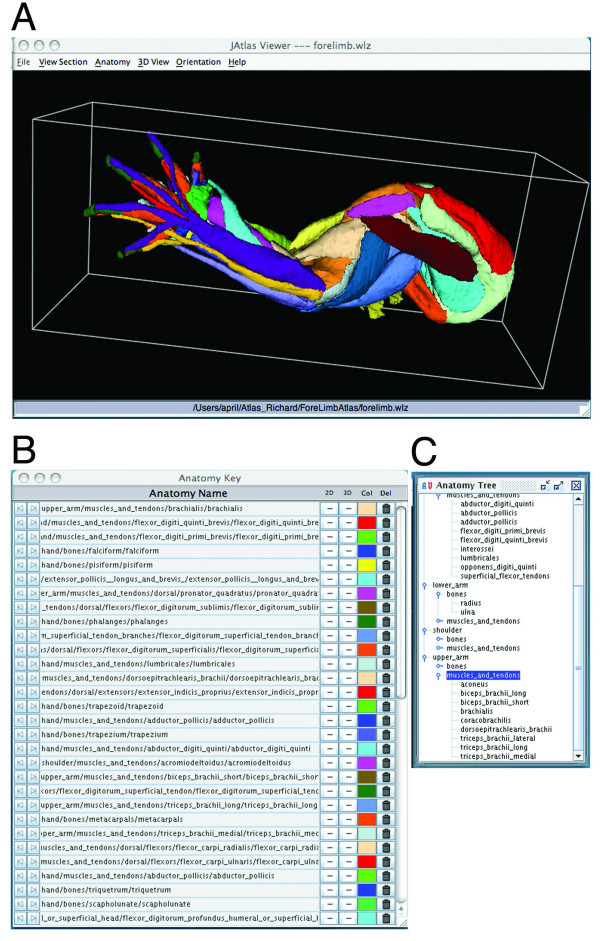
**JAtlasView.** A: JAtlasView showing an interactive 3D model of the muscles, tendons and bones of the embryonic mouse forelimb at E14.5. B: Anatomy key. Shows the name of each structure in the 3D model, its ontogeny and colour code. Structures can be switched off/on in the viewer window by clicking on the '+/-' icon in the Anatomy Key. C: Anatomy Tree. Shows the ontogeny of structures. Names can be clicked so structures appear in the 3D model.

**Figure 2:**
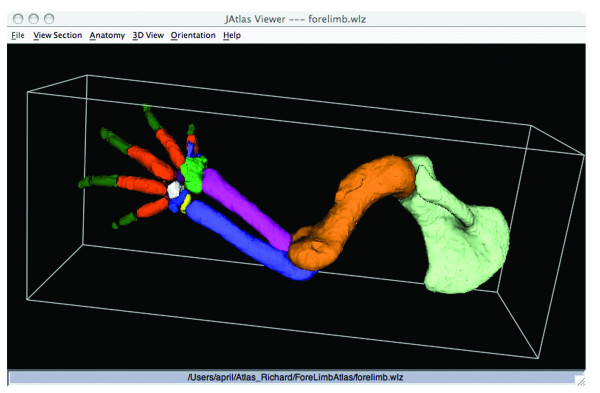
JAtlasView showing an interactive 3D model of the skeletal structures of the embryonic mouse forelimb at E14.5 after switching off muscles and tendons in the Anatomy Key.

The forelimb and hindlimb datasets and JAtlasView are available as free downloads accessible from our webpage (see Availability and Requirements section). The datasets include Woolz (.wlz) and Visualisation Toolkit (.vtk) format files in a structured folder hierarchy (reflecting the anatomy) for generating the stack of reconstructed images and segmented structures of the limb. The webpage includes instructions for downloading datasets and software and instructions on how to use the Atlas (Additional file 1). First, the forelimb.wlz or hindlimb.wlz data set is opened in JAtlasView (Fig. 1A). The 'Anatomy Tree' is then loaded, showing the ontology of structures (Fig. 1C). Structures are organized by region of the limb (ie. hand, lower arm), with sub-categories for muscles and bones. By clicking on the names of structures, users can build a 3D model showing selected muscles, tendons, and bones of the limb as separate coloured objects. Rotation, translation, and magnification of the model are controlled by keyboard or mouse buttons. The 'Anatomy Key' shows the name of each structure in the 3D model, its full name in the structure hierarchy and colour code (Fig. 1B). The colour of an object can be changed by clicking on the coloured box in the Anatomy Key. To switch off/on the object in the 3D view, the '+/-' icon on the Anatomy Key is clicked. For example, if the user wanted to only see the skeleton of the limb, muscles can be switched off (Fig. 2). JAtlasView runs as a Java Network Launching Protocol (.jnlp) program that connects to a host server at HGU-MRC. Updates to the software will be provided, so that the latest version is downloaded. After the first time the .jnlp program is launched, a version of the software is cached on the computer and can run off-line.

### Viewing 2D models

**Figure 3:**
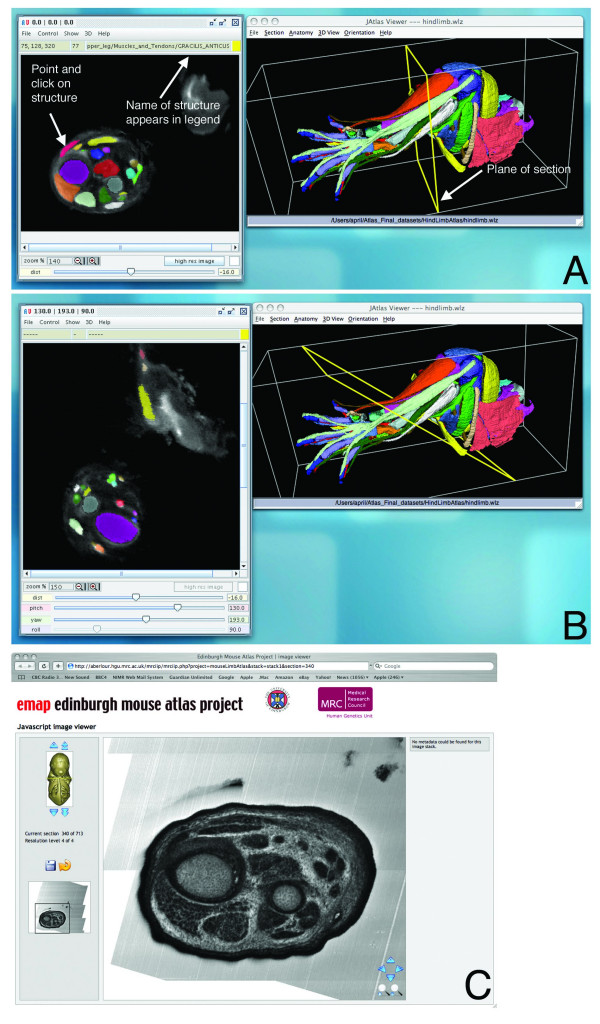
**JAtlasView showing 2D section views through the hindlimb.** A: XY section view. Using the point-and-click function of the 2D viewer, structures can be clicked on in the section and the name appears at the top of the 2D window. The plane of section is visible in the 3D view. B: A user-defined section. The plane of section is changed by adjusting 'yaw' and 'pitch'. C: High-resolution section viewer. By clicking on "high-res image" in the 2D viewer, the Java Section Viewer is launched. Users can move arrows to pan through the stack, magnify and translate sections. This section is equivalent to the section shown in 3A.

The 3D model is linked to a series of 2D sections in orthogonal XY, XZ, and YZ, or user-defined axes under "View Section" in JAtlasView. If structures are switched on in the 3D view, they are defined in the section view (Fig. 3A). The name of an undefined structure in a section can be determined by selecting "Mouse Click Anatomy" under "Show" in the 2D window. Then, when a structure is clicked on in the 2D view, its name will appear at the top of the 2D window (Fig. 3A). A scroll bar allows panning through the stack, and the plane of section is visible in the 3D view (Fig. 3A and 3B). To switch off/on the object in the 2D view, click on the '+/-' icons on the Anatomy Key. For user-defined sectioning, "Control" and "rotation" on the 2D viewer allows changes to the "yaw" and "pitch" of the section (Fig. 3B). To see HREM sections of a stage-matched E14.5 forelimb or hindlimb, the "high-res image" button in the 2D viewer (Fig. 3C) links to a Java-based Section Viewer program showing an HREM section equivalent (but not identical) to the OPT-generated section in the 2D viewer. Users can move arrows to pan through the stack, magnify and translate sections. Although the HREM section series is not annotated, by comparing sections with the annotated 2D series users can find the names of structures.

## Utility

This resource is aimed for use by biologists interested in limb anatomy, although we encourage other members of the scientific and educational community to use it. As a 3D tool, the Atlas is useful for comparative anatomy of whole mount prepared samples, as a dissection aide and as an alternative to animal dissection. At present, most biologists studying limbs do not routinely have access to 3D imaging technology, but study 2D histological sections of limbs. The 2D section series of the Atlas allows the user to compare sections of mutant limbs with wild-type limbs. By adjusting the plane of section, users can find the exact plane of section of their histological sample (Fig. 3C). Using the point-and-click feature, users can quickly and easily find out the names of structures and see where in the 3D model the section is cut. As 3D imaging becomes a routinely used technology, we envisage that the Atlas will become an increasingly valuable resource for interpreting 3D data. Additionally, as computer-automated segmentation technology is continually improving [22,23], it will be possible in the future to rapidly segment 3D datasets of mutant limbs to make models that can be compared with wild-type references.

An objective of this project has been to provide a freely available resource, avoiding the accessibility issues involved in using some printed resources and commercial software. Since the nomenclature for limb structures can vary between printed references, an additional aim of this project is to provide a standardised naming system for limb structures. A glossary of forelimb and hindlimb muscle names used in the 3D database and how they may differ in other text references are available via the 3D Atlas website.

## Discussion and conclusion

The Mouse Limb Anatomy Atlas is a free, web-based, standardised reference of limb muscle, tendon and skeletal structures at embryonic day 14.5. The Atlas features interactive and annotated 2D and 3D models of the forelimb and hindlimb, showing over 60 individually segmented structures. This is the first complete reference tool for studying the embryonic mouse limb and the first 3D atlas of limb anatomy. This resource presents a novel, accessible, intuitive approach for studying mouse limb anatomy that will facilitate analysis of limb morphology and the characterisation of mutant limb phenotypes. We also expect it will be an excellent reference tool for a broad range of the scientific community and be a particularly useful educational tool.

We hope that as the Atlas is used, we will get feedback from users about any discrepancies in ontology with existing references and how features of the datasets and software can be improved. We plan to modify the 3D atlas where possible to increase its functionality. In the future, it will be possible to add datasets for different stages of development and models of other tissues of the limb, including the limb vascular and neural structures.

## Availability and requirements

• Project name: The Mouse Limb Anatomy Atlas.

• Project home page: 

• Forelimb and Hindlimb datasets downloads: 

JAtlasView download: 

• Operating system(s): Solaris, Linux, Mac OSX. The viewer can run successfully on Windows 2000 – Internet Explorer6, Windows 2000 (installed Java) – Internet Explorer6, Vista – Internet Explorer7, Windows XP – Internet Explorer6, Windows XP (installed Java) – Internet Explorer7 and Firefox 2.0.

• Other requirements: Java 1.4, Java 2 Platform Standard Edition Runtime Environment, Version 5.0 or later (J2RE)

• Any restrictions to use by non-academics: None.

## Authors' contributions

AD prepared, scanned, segmented the limb data, and reconstructed the HREM section series. NB and RB made improvements to JAtlasView software. RB converted datasets to Woolz and vtk formats. NB generated the Java Section Viewer dataset. DD made suggestions for implementing the Atlas and helped coordinate communication between NIMR and HGU-MRC. TM set-up the OPT scanning system and reconstruction software. MB performed the HREM sectioning. ML is the main supervisor for this project and the corresponding author for feedback of the on-line resource. All authors read and approved the final manuscript.

## Supplementary Material

Additional file 1 3dlimbatlasdemo. Quicktime movie demonstrating how to navigate and use the 3D limb anatomy atlas. Click here for file
